# Loss of Ubiquitin Carboxy-Terminal Hydrolase L1 Impairs Long-Term Differentiation Competence and Metabolic Regulation in Murine Spermatogonial Stem Cells

**DOI:** 10.3390/cells10092265

**Published:** 2021-08-31

**Authors:** Whitney F. Alpaugh, Anna L. Voigt, Rkia Dardari, Lin Su, Iman Al Khatib, Wisoo Shin, Taylor M. Goldsmith, Krysta M. Coyle, Lin A. Tang, Timothy E. Shutt, Claudia Klein, Jeff Biernaskie, Ina Dobrinski

**Affiliations:** 1 Department of Comparative Biology & Experimental Medicine, Faculty of Veterinary Medicine, University of Calgary, Calgary, AB T2N 4N1, Canada; wfalpaug@ucalgary.ca (W.F.A.); anna.voigt1@ucalgary.ca (A.L.V.); rdardari@ucalgary.ca (R.D.); lsu@ucalgary.ca (L.S.); wshin@ucalgary.ca (W.S.); taylor.goldsmith@recombinetics.com (T.M.G.); krysta.coyle@dal.ca (K.M.C.); linaileentang@gmail.com (L.A.T.); jabierna@ucalgary.ca (J.B.); 2 Departments of Medical Genetics and Biochemistry & Molecular Biology, Cumming School of Medicine, University of Calgary, Calgary, AB T2N 4N1, Canada; iman.alkhatib1@ucalgary.ca (I.A.K.); timothy.shutt@ucalgary.ca (T.E.S.); 3 Department of Molecular Biology & Biochemistry, Simon Fraser University, Burnaby, BC V5A 1S6, Canada; 4 Federal Research Institute for Animal Health, Friedrich-Loeffler-Institute, 31535 Neustadt, Germany; Claudia.Klein@fli.de

**Keywords:** UCH-L1, murine spermatogenesis, germ cell differentiation, metabolic regulation, mitochondrial capacity

## Abstract

Spermatogonia are stem and progenitor cells responsible for maintaining mammalian spermatogenesis. Preserving the balance between self-renewal of spermatogonial stem cells (SSCs) and differentiation is critical for spermatogenesis and fertility. Ubiquitin carboxy-terminal hydrolase-L1 (UCH-L1) is highly expressed in spermatogonia of many species; however, its functional role has not been identified. Here, we aimed to understand the role of UCH-L1 in murine spermatogonia using a *Uch-l1^−/−^* mouse model. We confirmed that UCH-L1 is expressed in undifferentiated and early-differentiating spermatogonia in the post-natal mammalian testis. The *Uch-l1^−/−^* mice showed reduced testis weight and progressive degeneration of seminiferous tubules. Single-cell transcriptome analysis detected a dysregulated metabolic profile in spermatogonia of *Uch-l1*^−/−^ compared to wild-type mice. Furthermore, cultured *Uch-l1^−/−^* SSCs had decreased capacity in regenerating full spermatogenesis after transplantation in vivo and accelerated oxidative phosphorylation (OXPHOS) during maintenance in vitro. Together, these results indicate that the absence of UCH-L1 impacts the maintenance of SSC homeostasis and metabolism and impacts the differentiation competence. Metabolic perturbations associated with loss of UCH-L1 appear to underlie a reduced capacity for supporting spermatogenesis and fertility with age. This work is one step further in understanding the complex regulatory circuits underlying SSC function.

## 1. Introduction

Mammalian spermatogenesis is a complex, highly regulated process of cell proliferation and differentiation sustained by spermatogonial stem cells (SSCs). These cells give rise to progenitors that undergo a finite number of synchronous cell divisions before entering meiosis as a spermatocyte [1]. Previously considered a cell population committed to differentiation, an early subset of progenitors can de-differentiate and regain stem cell capacity, which highlights the plasticity of the undifferentiated, mitotic germ cell state [2,3].

Clear identification of a definitive SSC has remained elusive, due to the lack of biochemical and molecular markers that unequivocally distinguish SSCs from other undifferentiated spermatogonia. Several putative SSC markers have been described, such as inhibitor of DNA binding 4 (*Id4*), paired box protein Pax-7 (*Pax7*), glial cell-line derived neurotrophic factors receptor alpha 1 (*Gfr*a*1*) and promyelocytic leukemia zinc finger protein (*Plzf*). However, germ cell populations that express these markers are generally regarded as a mixed population containing SSCs among other undifferentiated spermatogonia [4,5,6]. Unlike in other stem cells, tyrosine-kinase receptor *c-Kit* neither is expressed in SSCs in vivo nor plays a role in their maintenance, but it is critical to the primordial germ cells’ movement towards the basement membrane, where they give rise to SSCs [7]. A study showed, however, that c-KIT positive and c-KIT negative cultured germ cells displayed comparable SSC activity after transplantation [8]. On the other hand, stimulated by retinoic acid gene 8 (*Stra8*), which is the receptor for retinoic acid and a key molecule in initiating meiosis [9], also plays a role in SSC maintenance, proliferation and self-renewal [10].

The process of spermatogenesis through mitotic, meiotic, and post-meiotic phases is governed by several cellular processes. Evidence underlines the key role of ubiquitination in the derivation of SSCs and differentiation of spermatogonia from gonocytes [11], in genetic recombination [12] and in sex chromosome silencing during meiosis [13]. Ubiquitination is a post-translational modification that regulates several cellular processes such as protein turnover, DNA damage response and intracellular signaling [10,11,12], which can be reversed through the action of de-ubiquitinase enzymes [14].

Ubiquitin carboxy-terminal hydrolase 1 (UCH-L1), also known as PGP9.5, is classified as a de-ubiquitinase enzyme [15] that is primarily expressed in the nervous system and testes [15,16]. Beside its role in catalyzing the hydrolysis of ubiquitin from its target and thus preventing protein degradation [15], UCH-L1 can perform a variety of other functions, including binding and sequestering mono-ubiquitin, signaling for protein translocation within the cell, and stabilizing ubiquitin modifications [17,18].

The role of UCH-L1 in maintaining cell homeostasis under normal growth and oxidative stress conditions has been reported in vitro [19]. Evidence links the development of gracile axonal dystrophy [20,21], Parkinson’s and Alzheimer’s diseases to mutations or deletions in the *Uch-l1* gene [22,23,24,25]. Hussain et al. (2013) demonstrated the importance of UCH-L1 in regulating the balance between mechanistic Target of Rapamycin (mTOR) complexes by disrupting mTOR complex 1 (mTORC1) and promoting mTORC2 assembly [26], which plays a role in preventing brain degeneration and development of malignancies [27]. UCH-L1 deficiency, associated with enhanced mTORC1 activity, causes an early increased protein turnover, which leads to progressive neurodegeneration in *Uch-l1* deficient mice [28].

To date, attempts to describe the potential function for UCH-L1 in the testis are limited to a few studies of mutant testes that either carry a spontaneous in-frame deletion of *Uch-l1* [19] or overexpress *Uch-l1* in all tissues [29]. The testis of a mouse model that constitutively over-expresses UCH-L1 showed a meiotic block at the early pachytene spermatocyte stage and a reduction in proliferating spermatogonia, suggesting a role for UCH-L1 in spermatogonial proliferation and differentiation [29]. Conversely, the testes of a gracile axonal dystrophy (*gad*) mouse that had a spontaneous in-frame deletion of *Uch-l1* showed a decrease in proliferating cells, tubule size and cell numbers along with an increase in apoptosis and size of the remaining cells within the seminiferous tubules at 25 weeks of age [19]. Subsequent studies reported *gad* testes being resistant to cryptorchid-induced injury and early apoptotic first wave, suggesting a role in BAX/BCL-2 (BCL2 associated X, apoptosis regulator, B-cell lymphoma 2)-regulated apoptosis [30]. Yet the role of UCH-L1 and the site of the ubiquitination target in the testes remain to be further determined [20]. Loss of UCH-L1 does not result in compensatory up-regulation of other isoforms, suggesting a distinct, non-redundant cellular role of UCH-L1 [20].

Here we show that UCH-L1 is expressed in undifferentiated spermatogonia (SSC/progenitors) as well as in early differentiating spermatogonia. Taking advantage of the *Uch-l1^−/−^* mouse model that lacks all UCH-L1 function, we found an increased number of degenerative seminiferous tubules in *Uchl1^−/−^* compared to *Uch-l1^+/+^*, a condition that is preceded by a dysregulation of normal metabolic transitions in undifferentiated spermatogonia, as revealed by single cell RNA sequencing analysis. We further demonstrate a loss of differentiation competence of SSCs after transplantation in vivo and accelerated mitochondrial activity in vitro. These data point towards an important role of UCH-L1 in murine spermatogenesis. Loss of UCH-L1 is associated with reduced capacity of SSCs to support spermatogenesis and accompanied by increased oxidative phosphorylation (OXPHOS), mitochondrial dysfunction and metabolic perturbations. The disturbance of the tight regulation of normal metabolic transitions necessary for murine spermatogenesis associated with the loss of UCH-L1 could ultimately result in reduced fertility with age.

This work provides novel insight into the functional implication of UCH-L1 for the SSC metabolic homeostasis that is required for long-term metabolic plasticity and stem cell function.

## 2. Materials and Methods

### 2.1. Mice

*Uch-l1*^tm1Dgen^ (Deltagen Inc., Menlo Park, CA, USA), B6/SV129 and ROSA26 reporter line (B6.129S7-Gt(ROSA)26Sor/J) mice were obtained from the Jackson Laboratory. All animal experiments were carried out in compliance with the Canadian Council of Animal Care guidelines and approved by the University of Calgary’s Health Sciences Animal Care Committee (AC20-0058, Renewal 5 August 2020).

### 2.2. Cell Sorting and Culture

Spermatogonia were isolated from 6 to 8-day old *Uch-l1*^+/+^ and *Uch-l1*^−/−^ mice through magnetic-activated cell sorting (MACS; 18000, Stem Cell Technologies, Vancouver, BC, Canada) using a biotinylated rat anti-Thy1.2 mouse antibody (1:100, 553001, BD Biosciences, Mississauga, ON, Canada). Cells were incubated on ice for 30 min with gentle agitation, washed twice with PBS and incubated with Dynabeads^TM^ M-280 Streptavidin (1:200, 11205D, Thermo Fisher Scientific, Mississauga, ON, Canada). Thy1 positive cells were resuspended in a-MEM serum-free clusters medium supplemented with GDNF (40 ng/mL), GFRa1 (300 ng/mL) and β-FGF (1 ng/mL) and cultured as previously described [31,32]. Cells were collected on days 3 and 6 of passages 3, 5 and 8 for analysis. Cell counts and viability were determined using the Trypan Blue (T8154, Sigma, Oakville, ON, Canada) exclusion assay. Cell proliferation was assessed via EdU (5-ethynyl-2′-deoxyuridine) incorporation assay after a 6-hour pulse following the manufacturers’ instructions (C10086, Invitrogen Click-It EdU, Thermo Fisher Scientific).

### 2.3. Histology and Immunohistochemistry

*Uch-l1^+/+^, Uch-l1*^+/−^ and *Uch-l1*^−/−^ mice testis tissues were collected at post-natal days 0, 7, 17, 30, 60, 90 and 120, fixed in Bouin’s solution or 4% paraformaldehyde (PFA) and embedded in paraffin. Serial paraffin-embedded sections were prepared and stained with hematoxylin and eosin (H&E) for histology examination. Immunohistochemistry was performed on PFA-fixed embedded sections following deparaffination, rehydration and heat-induced antigen retrieval steps. The sections were incubated with primary antibodies against PLZF (HPA001499, Sigma,), LIN28A (8641, Cell Signalling Technologies, Danvers, MA, USA), UCH-L1 (13179, Cell Signalling Technologies) and SOX9 (sc-17341, Santa Cruz Biotehnologies, Dallas, TX, USA,), followed by an incubation with species-specific secondary Alexa Fluor 488, 555 or 647 antibodies. Nuclei were labeled with 4′,6-diamidino-2-phenylindole (DAPI) (VECTH1200, BioLynx, Brockville, ON, Canada). At least 50 cross-sections were processed per sample from 3 tissue sections 50 µm apart. For comparison purposes, PFA-fixed archival testis sections from other mammalian species—adult human, adult buck, adult cat, adult rat, adult sheep, juvenile porcine, juvenile goat, juvenile horse and juvenile monkey—were also processed and analyzed.

### 2.4. TUNEL Assay

The DeadEnd^TM^ Fluorometric TUNEL system (488) (G3250, Promega, Madison, WI, USA) was used to detect and quantify cell apoptosis in tissue sections. The labeling was performed according to the manufacturer’s recommendations and nuclei were stained with DAPI. The assay catalytically incorporates fluorescein-12-dUTP at 3′-OH ends of DNA, which allows direct visualization of labeled DNA (in green) through fluorescence microscopy.

### 2.5. Flow Cytometry

Single cells were collected from 17, 90 and 120-day mice testis tissue using a two-step enzymatic digestion. Briefly, tissues were digested with 6 mg/mL collagenase IV (LS004189, Worthington Biomedical Corporation, Lakewood, NJ, USA) for 15 min at 37 °C, followed by a 5-min incubation with 0.05% Trypsin-EDTA (59417C, Sigma) and 10 mg/mL DNase I (DN25, Sigma). Testicular cells from 90 to 120-day old mice were pelleted at 500 g for 5 min, washed twice with cold PBS and fixed for 30 min at 4 °C using BD Cytofix^TM^ Fixative Buffer (554655, BD Biosciences). Cells were washed with cold PBS, permeabilized for 30 min at 4 °C with BD Perm/Wash^TM^ buffer (554723, BD Biosciences), and blocked using PBS supplemented with 2% BSA and 0.1% NaN3 for 15 min at room temperature. Single testicular cells were stained with mouse Alexa 488-conjugated PLZF (53-9320, Thermo Fischer Scientific) and rat PE/Cy5-conjugated c-KIT (ab25495, Abcam, Waltham, MA, USA) antibodies and their respective isotype controls, mouse Alexa 488 conjugated IgG1 kappa (53-4714-42, Thermo Fisher Scientific) and rat PE/Cy5-conjugated IgG2b kappa (ab239465, Abcam), at room temperature for an hour. Cells were washed twice and analysed with a BD FACSClalibur™ Cell Analyzer instrument (J342976, BD Biosciences) and FlowJo software version 10 (BD Biosciences).

### 2.6. Quantitative PCR

RNA was isolated from 7, 17 and 120-day old mice testis and primary cultured cells using RNeasy Mini Kit (74106, Qiagen, Toronto, ON, Canada). RNA was reverse-transcribed into cDNA using ABI High Capacity cDNA synthesis kit (4368814, Fisher Scientific, Ottawa, ON, Canada) according to the manufacturer’s instructions. cDNA amplification was carried out in an ABI 7500 Fast qPCR machine (721BRO1344, Thermo Fisher Scientific) using Bio-Rad Sso Fast Eva Green master mix with low Rox (172-5212, Bio-Rad laboratories, Mississauga, ON, Canada). The qPCR results were baseline-corrected using LinReg PCR. Primer specificity was confirmed by sequencing RT-PCR amplified products and melt curve analysis (Primers list: Table S1).

### 2.7. Germ Cell Transplantation

To enable the identification and quantification of cells and colonies transplanted into a germ cell-depleted recipient testis, Uch-l1^−/−^ mouse (B6;129P2-Uchl1tm1Dgen/Mmnc) was crossed with a ROSA26 reporter line (B6.129S7-Gt(ROSA)26Sor/J) expressing the lacZ transgene. Spermatogonia collected from Uch-l1^+/+^, Uch-l1^−/−^ or heterozygous gtROSA 6 to 8-day old pups, which ubiquitously express the LacZ reporter gene, were cultured for 3 to 5 passages prior to transplantation. Approximately one million cells contained in 8 μL were transplanted into six-week old B6/SV129 mice via the efferent duct during week 6 after busulfan treatment (50 mg/kg) (B2635, Sigma) as previously described [31]. Three independent cell preparations per genotype were used for transplantation. Testes from recipient B6/SV129 males were collected two and 10 months post-transplantation. To visualize donor-derived spermatogenesis, testes were fixed in 4% PFA for 2 h at 4 °C, incubated with X-gal substrate at 4 °C overnight, then kept for another 2–4 h at 37 °C until the donor-derived cells turned deep blue. Colonies were counted and individual colony length measured. The blue intensity of colonized tubules was qualitatively scored against an open reference of three color intensities (light, medium and dark blue). Each colony was measured for the percentage that was light, medium and dark blue. Seminiferous tubules were collected, fixed in Bouin’s solution for 2 h at room temperature, processed and embedded in paraffin. The embedded sections were used for histological analysis of donor-derived colonies.

### 2.8. Oxygen Consumption Rate (OCR)

OCR assays were performed using a XF24 Seahorse flux analyzer according to the manufacturer’s instructions (Seahorse Bioscience, North Billerica, MA, USA). Plates were treated with 250 μL (100 μg/mL) poly-D-lysine (Corning^®^ BioCoat™; 354210, Corning, Glendale, AR, USA) for 30 min. A total of 15 × 10^6^ cells was immobilized to a monolayer with centrifugation at minimal acceleration to 45 G and directly stopped with zero breaking. The cell medium was topped up to 500 μL and the plate was subsequently centrifuged with reverse plate orientation. Respiration (OCR) was measured with an OCR assay medium (XF DMEM; 102365-100, Agilent, Santa Clara, CA, USA) supplemented with 5.56 mM glucose, 1 mM pyruvate and 4 mM glutamine. The experiment was performed with oligomycin (2 μM; BML-CM111, Enzo Life Sciences, Farmingdale, NY, USA), mitochondrial uncoupling compound carbonyl cyanide 4-(trifluoromethoxy) phenylhydrazone FCCP (2 μM; BML-CM120, Enzo Life Sciences), respiratory chain inhibitor antimycin A (A8674, Sigma Aldrich) and rotenone (1 μM each; ALX-350-360, Enzo Life Sciences) [33]. All Seahorse results were normalized to the number of cells per well (15 × 10^4^ cells/0.32 cm^2^).

### 2.9. Single-Cell RNA-Sequencing

Testicular cells were collected via enzymatic digestion from two 8-week old mice from each genotype. Cells were stained with goat anti GFR⍺1 protein (GT15004, Neuromics, Edina, MN, USA) and rat anti c-KIT protein (ab65525, Abcam) and subjected to fluorescence-activated cell sorting (FACS) to enrich for spermatogonia using a FACSAria III (BD Biosciences). Briefly, previous experiments have identified a population of cells based on light scatter properties that is enriched in spermatogonia (Figure S1A). All c-KIT+ and GFRa1+ cells (c-KIT+ GFRa1-, c-KIT+ GFRa1+, and c-KIT-GFRa1+) are within this spermatogonia-enriched cell population (Figure S1B–D). Isolated primary spermatogonia samples were resuspended in PBS supplemented with 1% BSA and 6000–10,000 cells per sample were processed using Chromium^TM^ Single Cell 3′ v2 Chemistry library prep kit according to the manufacturer’s instructions (10X Genomics, San Francisco, CA, USA). Samples were sequenced on an Illumina HiSeq4000 with paired end 50 base pair reads by Genome Quebec (Montreal, QC, Canada). The raw counts were subsequently processed with a 10x Genomics CellRanger pipeline using default settings, after which the outputs from the four individual samples were combined using the CellRanger aggr function to normalize the sequencing depth and produce aggregated gene-cell UMI count matrix. A total of 7874 single germ cells (spermatogonia to spermatids) was used for the downstream analysis.

### 2.10. Single Cell RNA Sequencing Analyses

The concatenated gene-cell barcode matrix was imported into Seurat3.1.0 [34]. A pre-filtration step to remove low quality cells was performed and only cells expressing ≥ 200 detected genes and genes expressed in ≥ 3 cells were kept for the downstream analysis. The individual samples were then subjected to the standard Seurat pre-processing workflow to select and filter high quality cells based on the QC metrics (genes detected, UMI counts and mitochondrial genes). To perform a comparative analysis between the two genotypes (*Uch-l1^−/−^* vs. *Uch-l1^+/+^*), Seurat V3 integration workflow was used to merge, correct for the batch effect and generate a new integrated Seurat matrix that was used for further analysis. To identify the dimensionality of the dataset, the “*ElbowPlot*” function was applied, and the first 15 Principal components (PCA) were selected to run the “*FindNeighbors*”, “*FindClusters*” and “*RunUMAP/RunTSNE*” functions with a resolution of 0.5. Once clusters were generated, the “*FindMarkers*” function was applied to identify differentially expressed genes (DEG) with *p* values lower than 0.01 and fold change of 1 or higher. The “*VlnPlot*”, “*FeaturePlot*”, and “*Dimheatmap*” functions were used to visualize genes of interest.

### 2.11. Gene Set Enrichment Analysis (GSEA)

GSEA was performed with the javaGSEA application (version 4.0.3) available online (javaGSEA. http://www.broadinstitute.org/gsea/downloads.jsp, accessed on 24 August 2021) with default settings [35] (http://software.broadinstitute.org/gsea/, accessed on 24 August 2021). A pre-ranked list consisting of DEGs and their corresponding fold-change values was used as an input to determine the enrichment scores for pathways. The curated gene sets used for reference were downloaded from Molecular Signatures Database (MSigDB) [36]. The enriched pathways were displayed as a heat map using the *“heatmap.2”* function in R studio. The gene network was built with STRING [37].

### 2.12. Statistical Analysis

Quantitative data are displayed as mean + standard deviation. Non-parametric *t*-tests (Mann–Whitney U tests) were performed when comparing two data sets and Kruskal–Wallis ANOVA for three data sets according to statistical guidelines. Significance was set at *p* < 0.05. For the Seahorse experiment, the results are presented as mean ± SEM, if not otherwise stated. Normality was tested with Shapiro–Wilk test (if possible, by sample size via Anderson–Darling test, D’Agostino–Pearson test and Kolmogorov–Smirnov test); normality was assumed if all performed tests indicated a normal distribution. Normally distributed data was analyzed with an unpaired parametric *t*-test, otherwise a non-parametric *t*-test (Mann–Whitney test) was used to compare ranks. Unequal standard deviation was accounted for with Welch’s correction. A value of *p* < 0.05 was considered statistically significant. GraphPad Prism 8.0 software was used for all statistical analyses.

## 3. Results

### 3.1. UCH-L1 Is Expressed in Mouse Spermatogonia and Sertoli Cells

**Figure 1:**
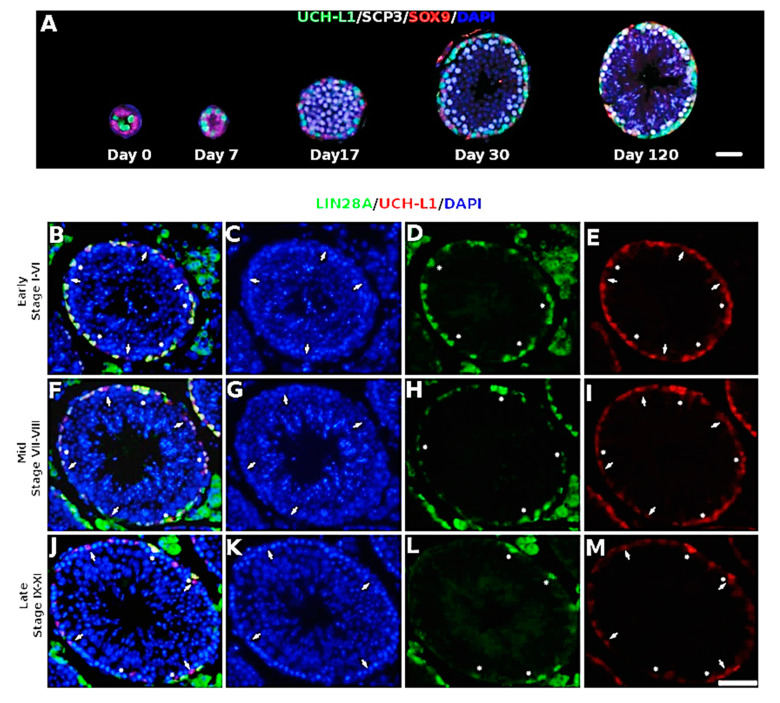
UCH-L1 expression in mouse testis. (**A**) Immunohistochemistry panel of UCH-L1, SOX9, SCP3 and DAPI at 0, 7, 17, 30 and 120 days old. Scale bar = 50 µm. (**B**–**M**) Immunohistochemistry panel of UCH-L1, LIN28A and DAPI. Arrows indicate Sertoli cells, determined by nuclear morphology, and stars indicate LIN28A positive cells. Scale bars = 50 µm.

To examine the expression pattern of UCH-L1 in testes, immunohistochemistry was performed on PFA-fixed, paraffin-embedded sections from *Uch-l1^+/+^* mouse testes collected at different stages of development. As in other mammalian species, UCH-L1 in mice is expressed in cells located along the basement membrane of seminiferous tubules but not in further differentiating germ cells (Figure 1; Figure S2). Figure 1A illustrates the expression of UCH-L1 and SRY-box 9 (SOX9) from day 0 to day 120, along with the expression of synaptonemal complex protein (SPC3), a marker specifically expressed during meiosis in spermatocytes. At day 30, the expression of UCH-L1 was detected in Sertoli cells as well (Figure 1B–M), which is an expression specific to rodents [38]. In addition, UCH-L1 is co-expressed with LIN28A, a marker largely expressed in the spermatogonial progenitor population [39]. All LIN28A positive spermatogonia were UCH-L1 positive and all UCH-L1 positive cells that were not Sertoli cells were LIN28A positive (Figure 1B–M).

### 3.2. Uch-l1^−/−^Mice Have Decreased Testis Weight and Increased Degeneration of Seminiferous Tubules

**Figure 2:**
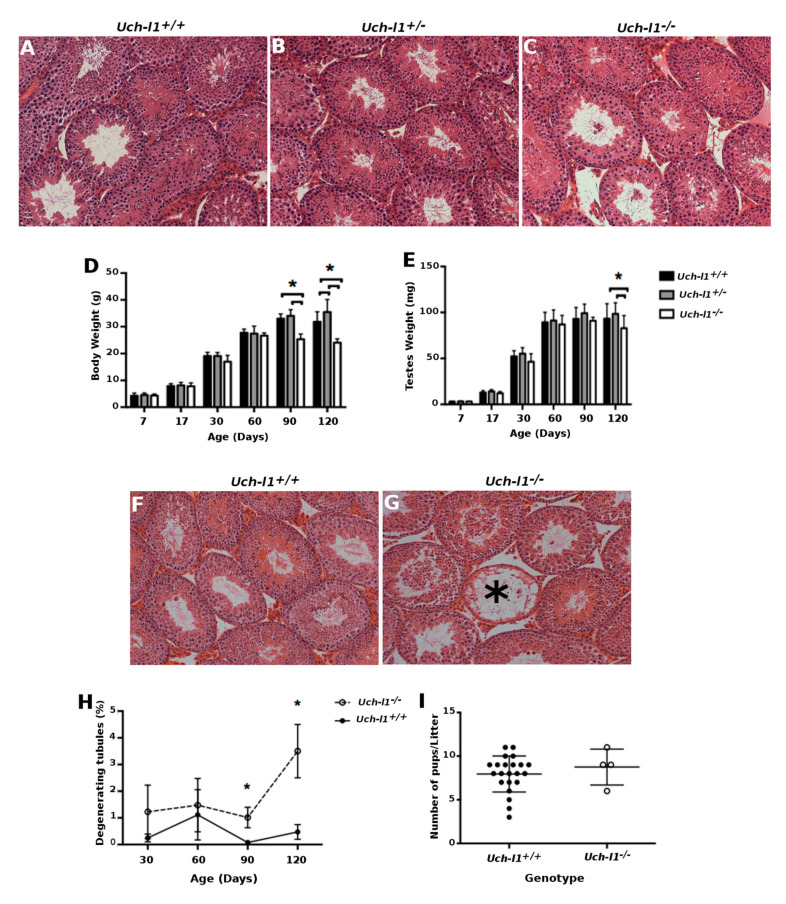
Phenotype of *Uch-l1*^+/+^, *Uch-l1^+/−^* and *Uch-l1^−/−^* testes. (**A**–**C**) H&E histology images of *Uch-l1*^+/+^, *Uch-l1^+/−^* and *Uch-l1^−/−^* testes at 120 days old. Scale bar = 200 µm. (**D**) Body weight of *Uch-l1*^+/+^, *Uch-l1^+/−^* and *Uch-l1^−/−^* mice. (**E**) Weight of *Uch-l1*^+/+^, *Uch-l1^−/−^* and *Uch-l1^−/−^* testes. (**F**,**G**) 120-day old *Uch-l1*^+/+^ and *Uch-l1*^−/−^ testis histology, respectively. Star indicates a degenerating seminiferous tubule. Scale bar = 200 µm. (**H**) Percentage of degenerating seminiferous tubules at 30, 60, 90 and 120 days of age in *Uch-l1*^+/+^ and *Uch-l1*^−/−^ testes. N = 6/genotype/time point. (**I**) Litter size from *Uch-l1*^+/+^ and *Uch-l1*^−/−^ male matings to *Uch-l1*^+/+^ females. Individual data points represent one litter. * indicates significant differences (*p* < 0.05).

To determine whether UCH-L1 deficiency affects the morphology and cell composition of the seminiferous tubules, we histologically examined testes collected from *Uch-l1^+/+^, Uch-l1* ^+/−^ and *Uch-l1^−/−^* mice at different time points of testis development. Due to the strong neurodegenerative phenotype of the *Uch-l1^−/−^* mice, 120 days of age was ethically dictated as a final time point. In 7-day-old mice, only spermatogonia were detected in the seminiferous epithelium, followed by the first wave of spermatogenesis, with the appearance of primary spermatocytes at day 17 of age. By day 30, the first wave of spermatogenesis was completed and the 60, 90 and 120-day time points captured subsequent spermatogenic waves. Testes generally appeared histologically normal and achieved full spermatogenesis regardless of the genotype (Figure 2A–C).

However, a significant difference in body weight and testis weight was detected at days 90 and 120 between the two genotypes, while they were indistinguishable at earlier stages. The body weight of 90 and 120-day old *Uch-l1^−/−^* males was significantly lower than *Uch-l1^+/−^* and *Uch-l1*^+/+^ males, which was reflective of their progressive neurodegeneration (Figure 2D). There was also a significant decrease in testis weight at 120 days of age in *Uch-l1^−/−^* compared to *Uch-l1^+/−^* and *Uch-l1*^+/+^ genotypes (Figure 2E). Furthermore, changes in testicular weight were consistent with an increase in the number of degenerative seminiferous tubules in *Uch-l1^−/−^* compared to *Uch-1l*^+/+^ testes at days 90 and 120 of age (Figure 2F–H).

To assess whether these morphological defects impacted the ability of *Uch-l1* knockout mice to reproduce, *Uch-l1^+/+^* and *Uch-l1^−/−^* males at 2–3 months of age were mated with wild-type females. There was a significantly lower number of confirmed matings in the *Uch-l1^−/−^* males compared to *Uch-l1^+/+^*, which may indicate an issue with copulation (Figure 2I). However, the average size of the few *Uch-l1^−/−^* litters produced remained normal with healthy pups. The progressive decrease in testis weight together with an increase in the degeneration of seminiferous tubules may impact the fertility potential in *Uch-l1^−/−^* mice as they age.

**Figure 3:**
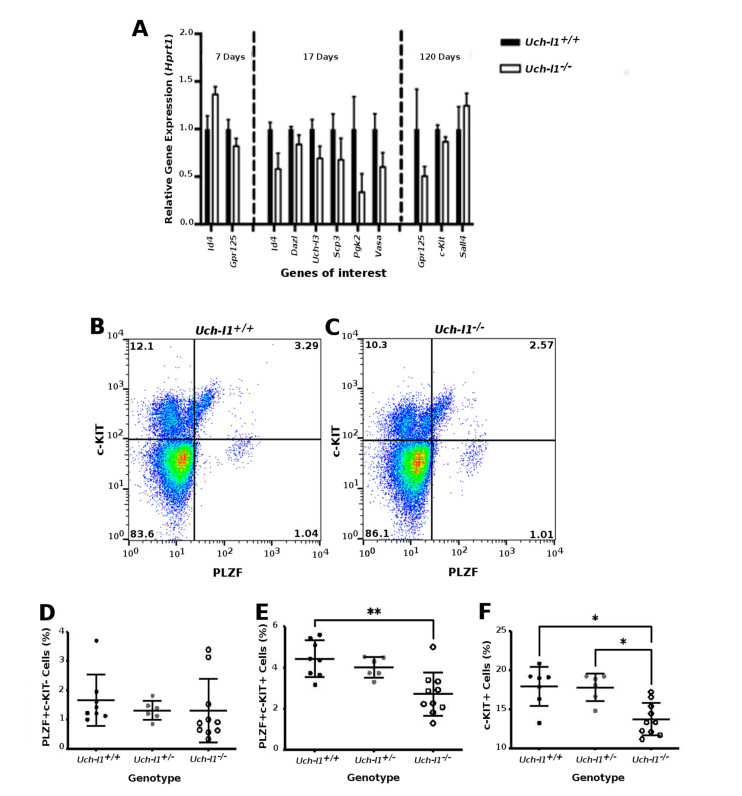
Analysis of whole *Uch-l1*^−/−^, *Uch-l1*^+/−^ and *Uch-l1*^+/+^ testes. (**A**) Quantitative PCR results of significantly differentially expressed genes in 7, 17 and 120-day old of *Uch-l1*^−/−^ relative to *Uch-l1*^+/+^ testes (**B**,**F**) Flow cytometry analysis of PLZF and c-KIT positive cells in *Uch-l1*^+/+^, *Uch-l1*^+/−^ and *Uch-l1*^−/−^ testes at 120 days of age. *n* = 6/genotype. (**B**,**C**) Representative dot plot of c-KIT and PLZF positive cells in *Uch-l1*^+/+^ and *Uch-l1*^−/−^ testes, respectively. (**D**) Percentage of PLZF+ c-KIT- cells in *Uch-l1*^+/+^, *Uch-l1*^+/−^ and *Uch-l1*^−/−^. (**E**) Percentage of PLZF+ c-KIT+ cells in *Uch-l1*^+/+^, *Uch-l1*^+/−^ and *Uch-l1*^−/−^. (**F**) Percentage of c-KIT+ cells in *Uch-l1*^+/+^, *Uch-l1*^+/−^ and *Uch-l1*^−/−^. * indicates significant differences (*p* < 0.05), ** represents significant differences *p* < 0.01.

As the only cells detected in degenerated seminiferous tubules were spermatogonia and Sertoli cells, we used the TUNEL assay on testes sections and qPCR for *Bcl2* and *Bax* gene expression to assess if cell apoptosis may account for the loss of differentiated germ cells. No difference was observed in the number of TUNEL-positive cells per seminiferous tubules (Figure S3A–D) or in the *Bcl2*/*Bax* ratio between the two genotypes at any given time point. First, our data do not corroborate the implication of UCH-L1-induced apoptosis during the first wave of spermatogenesis, as previously described in the *gad* mouse model. The *Uch-l1^−/−^* mouse model used in our study did not show any resistance to early wave apoptosis during the pre-pubertal stage of development, which is essential for functional spermatogenesis in adults [30]. Second, our results might be suggestive of a decrease in differentiation competence of *Uch-l1^−/−^* germ cells, rather than a loss of germ cells by apoptosis, since no difference was observed in cell apoptosis between the two genotypes at 120 days. Differences were also detected in relative expression of germ cell associated genes reflective of differentiation status during development (Figure 3A). At 7 days, a significant increase in stem cell-associated gene expression for *Id4* and a decrease in *Gpr125* gene expression was present in *Uch-l1*^−/−^ compared to *Uch-l1*^+/+^ testes. At 17 days old, there was an overall decrease in gene expression associated with stem cells (*Id4*) as well as differentiating germ cells (*Dazl*, *Uch-l3*, *Scp3*, *Pgk2*, *Vasa*). At 120 days, a significant increase was detected in progenitor cell differentiation-associated *Sall4* gene expression, associated with a significant decrease in differentiation-associated *c-Kit* expression in *Uch-l1*^−/−^ compared to *Uch-l1*^+/+^ testes (Figure 3A). These observations were further supported by the flow cytometry analysis (Figure 3B,C) showing fewer PLZF and c-KIT positive cells in *Uch-l1^−/−^* testis cells (Figure 3D–F).

### 3.3. Single Cell RNA Sequencing Analysis Reveals Similar Path Reflecting Stages of Spermatogenesis in Both Genotypes

**Figure 4:**
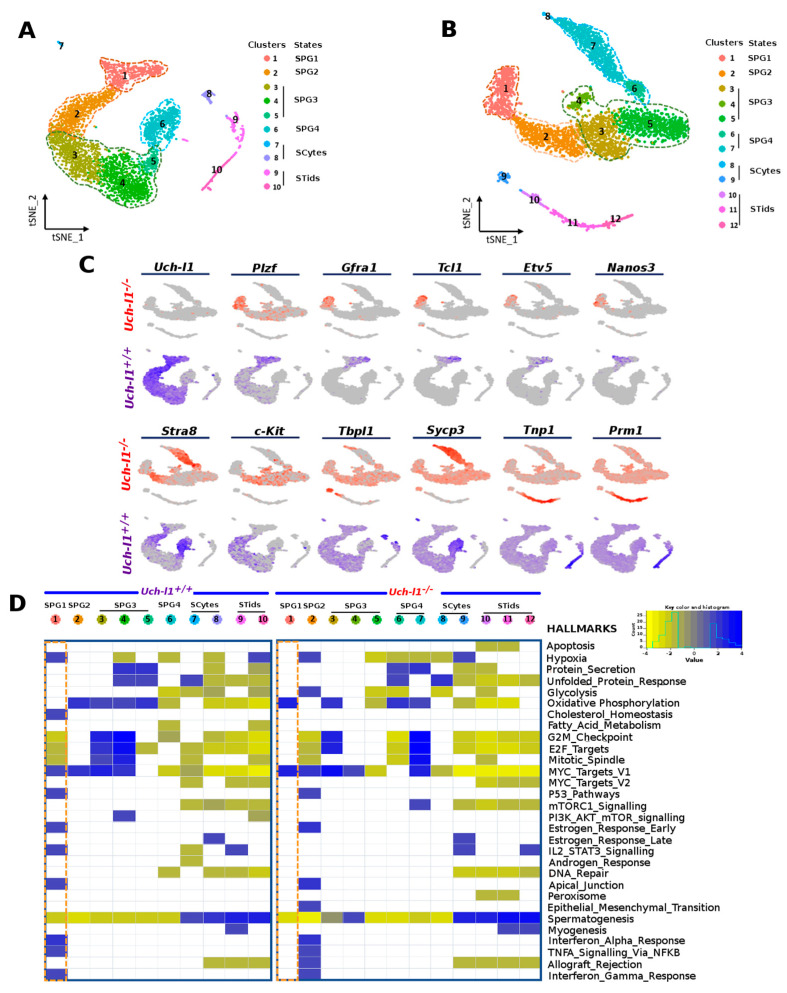
Single-cell RNA sequencing analysis of *Uch-l1*^+/+^ and *Uch-l1*^−/−^ spermatogonia. t-SNE analysis plot of single transcriptomes of *Uch-l1*^+/+^ (**A**) and *Uch-l1*^−/−^ (**B**), showing unbiased cell clusters distinguished by color according to the key. (**C**) The feature plot shows the expression of some genes that allow the distinction between spermatogonia, spermatocytes and spermatids. (**D**) Heatmaps of the upregulated and downregulated gene sets signatures determined by GSEA in *Uchl1*^+/+^ and *Uch-l1*^−/−^.

To capture any molecular changes that may emerge in germ cells due to the absence of *Uch-l1*, single-cell RNA sequencing was performed on 5000–8000 sorted germ cells (GFRa1^+^/c-KIT^+^ cells) collected from two *Uch-l1^−/−^* and two *Uch-l1^+/+^* mice. Seurat integrated analysis workflow was first used to merge the replicates from each genotype, correct for batch effect and perform the downstream analysis. Unsupervised, unbiased clustering projected onto t-distributed stochastic neighbor embedding (tSNE) revealed 10 and 12 cell clusters for *Uch-l1^+/+^* (Figure 4A) and *Uch-l1*^−/−^ (Figure 4B), respectively. In both genotypes, cell clusters were arranged in a continuous path that reflected different stages of spermatogenesis, including spermatogonia (SPG), spermatocytes and spermatids. SPG was further divided into four cell states, previously identified in different studies [40,41,42,43] and species [44]. The differentially expressed genes (DEG) that distinguish between these clusters in both genotypes are depicted in supplemental Figure S4A,B. As shown in Figure 4C, SPG-1 that encompassed SSC and progenitors were characterized by the expression of *Gfra1*, *Tcl1*, *Etv5*, and *Nanos3* and lack of expression of differentiation markers. SPG-2, identified as early differentiating cells, expressed both *c-Kit* and *Stra8* and lacked meiotic gene expression. SPG-3, which was further divided into three sub-clusters, were *c-Kit*^+^/*Stra8^−^*, and expressed the meiotic gene *Sycp3* at low levels. Cells in SPG-4 were *c-Kit^−^*/*Stra8^+^* and expressed high levels of *Sycp3* [40,41,42,43]. Of note, SPG-4 was organized into two distinct clusters in *Uch-l1^−/−^*, while it showed only one cluster in *Uch-l1^+/+^*. Although the number of cells present in clusters 7, 8, 9, 10, 11 and 12 in both genotypes was low, the analysis showed a clear distinction between the early and late meiotic process (spermatocytes and spermatids). Overall, there was no difference between the two genotypes in the expression of genes that mark SSC/progenitors, differentiating and differentiated cells. However, this assessment represents only a snapshot of a single time point, which might not represent further phenotypic heterogeneity that can emerge due to UCH-L1 deficiency with aging.

### 3.4. Single-Cell RNA Sequencing Analysis Shows an Altered Profile of Metabolic Transitions in Uch-l1^−/−^ SSC/Progenitors Populations

To determine whether germ cells displayed any functional change that would precede or accompany the degenerative phenotype of the seminiferous tubules, we used GSEA to identify enriched pathways whose genes were differentially expressed between cell clusters. Heat maps that display the enriched pathways for the two genotypes are shown in Figure 4D. The data showed an upregulation of gene sets associated with OXPHOS and *cMyc* signaling pathways in *Uch-l1^−/−^* SPG1, while eight gene sets were significantly upregulated in *Uch-l1^+/+^* SPG-1, including hypoxia, *p53* and *cMyc* signaling pathways. These data suggest the knockout of *Uch-l1* caused the SSCs/progenitors (SPG1) to shift their metabolism towards an oxidative metabolism as compared to the wild-type. Interestingly, the early-differentiating cell population (SPG2) showed an abnormal increase of glycolytic and hypoxic response-related genes in *Uch-l1^−/−^*, while there was an upregulation of OXPHOS-related genes in the wild type. This indicates an imbalance of normal metabolic transition after the loss of UCH-L1 in murine germ cells.

An enrichment of gene sets related to a higher cell proliferative state, namely G2M checkpoint, E2F_targets, and mitotic spindle, was observed in SPG3 from both genotypes. However, gene sets associated with the unfolded proteins pathway were upregulated in the late differentiating state (SPG4) in the knockout, while they were enriched early in the SPG3 population. The unfolded protein response (UPR) is a conserved cell process, induced in response to an accumulation of unfolded or misfolded proteins in the endoplasmic reticulum [45], which plays a role in cell homeostasis [46].

**Figure 5:**
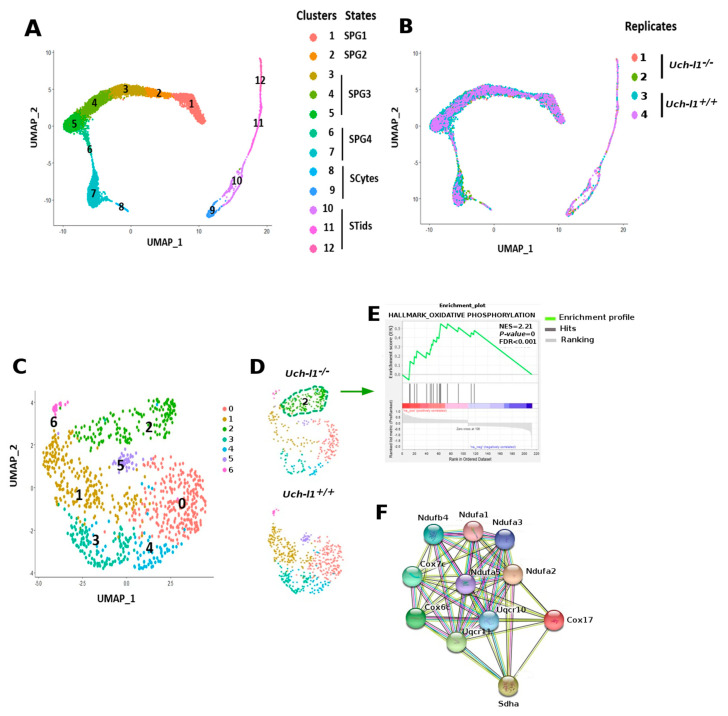
Integrative analysis across *Uchl1*^+/+^ and *Uch-l1*^−/−^ undifferentiated spermatogonia transcriptomes. (**A**) UMAP analysis plot of single transcriptome of four merged samples (2 *Uch-l1*^+/+^ and 2 *Uch-l1*^−/−^), showing unbiased cell clusters distinguished by color according to the key. (**B**) UMAP analysis showing individual samples grouped by color. (**C**) UMAP analysis plot showing the SPG1 (cluster 1) sub-clustered into seven distinct clusters (0 to 6). (**D**) UMAP analysis plot showing the two genotypes *Uchl-1*^+/+^ and *Uch-l1*^−/−^, separately. (**E**) GSEA showing enrichment of OXPHOS pathway in cluster 2 in *Uch-l1^−/−^*. (**F**) Gene network of 11 genes contributing to the enrichment score of OXPHOS.

To further examine whether the difference observed in enriched pathways in SPG1 was not due to batch effects between the two genotypes, and to achieve higher statistical power for differential expression analysis of SSC/progenitors, Seurat integrated analysis workflow was used to merge the four replicates (two from each genotype), correct for batch effect and perform the downstream analysis. Unsupervised clustering grouped the four transcriptomes into 12 distinct clusters, as illustrated by Uniform Manifold Approximation and Projection (UMAP) (Figure 5A,B). As expected, the clusters were organised into a continuous path as described above, showing the presence of four states of spermatogonia (SPG1-4), spermatocytes and spermatids (Figure 5A, Figure S4C). When extracted and reanalysed with a higher resolution, SPG1 showed further subdivision into seven individual clusters (Figure 5C,D), where both genotypes contributed to clusters 0, 1, 3, 4, 5 and 6, while cluster 2 was exclusively represented by the *Uch-l1^−/−^* genotype in one replicate (Figure S5A). Comparing the expression of some SSC/progenitor markers between the knockout and wild-type showed no differences, except for *Dmrt1* (Figure S5B). DMRT1 plays a role in maintaining the SSC pool under normal conditions and allowing progenitors to help restore the SSC pool when the germ cells are depleted [47]. The significantly identified DEGs in *Uch-l1^−/−^* (as compared to *Uch-l1^+/+^*) were loaded into GSEA to search for the enriched gene sets. Here again we confirmed the upregulation of gene sets related to OXPHOS in cluster 2 of SSCs/progenitors (Figure 5E), which was found only in the knockout. The 11 genes that significantly contributed to the enriched score of OXPHOS are presented as a network in Figure 5F. Collectively, these data suggest that UCH-l1 deficiency promotes a shift towards an oxidative metabolism in undifferentiated spermatogonia and a loss of normal metabolic transitions during SSC maintenance and differentiation [37,39,45].

### 3.5. Cultured Uch-L1^−/−^ SSCs Show a Decreased Capacity for Regeneration of Full Spermatogenesis

The neurodegenerative phenotype of the *Uch-l1^−/−^* mice limited the in vivo examination of spermatogonia to 120 days. Additionally, the expression of UCH-L1 in Sertoli cells in adult mice precludes interpretation of differences found between the *Uch-l1^−/−^* and *Uch-l1^+/+^* testes, as it cannot be unequivocally determined if any observed phenotype was due to the absence of UCH-L1 in Sertoli cells or spermatogonia. Therefore, transplantation of *Uch-l1^+/+^* and *Uch-l1^−/−^* spermatogonia into syngeneic mice was used to overcome these limitations.

First, we examined the impact of *Uch-l1* knockout on the growth and survival of spermatogonia in vitro. Testicular cells isolated from *Uch-l1^+/+^* and *Uch-l1^−/−^* 6 to 8-day old mice were maintained in culture for several passages and then collected for analysis on days 3 and 6 from passages 3, 5 and 8 (Figure S6A,B). Cell number, viability and proliferation were not significantly different between the two genotypes (Figure S6C,D). Gene expression for several spermatogonia-associated genes (*Id4*, *Plzf*, Lin28a, *c-Kit* and *Stra8*) was similar between genotypes (Figure S6E).

**Figure 6:**
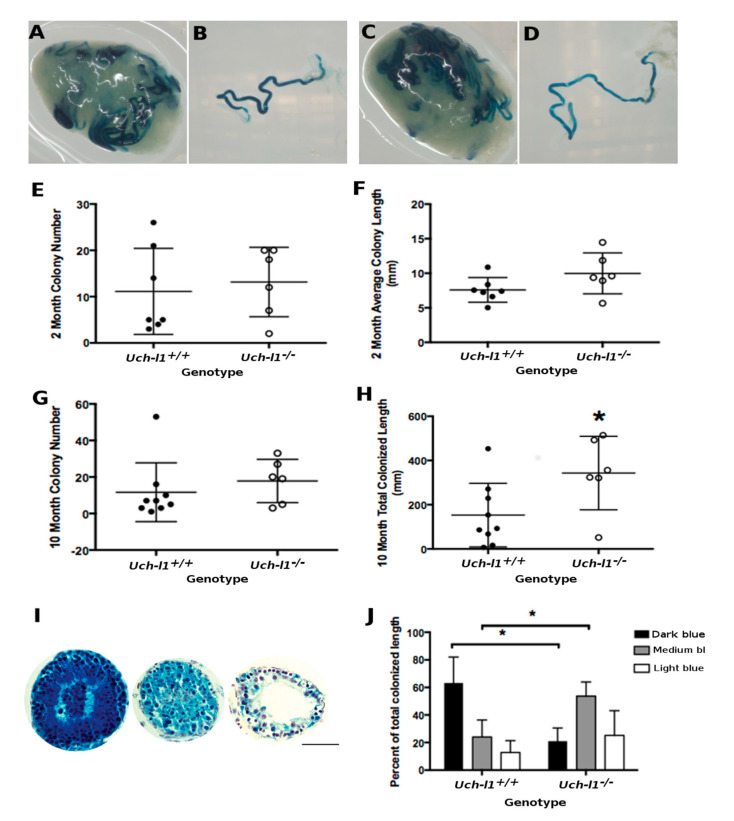
Transplantation of *Uch-l1*^+/+^ and *Uch-l1*^−/−^ SSCs. (**A**) Recipient testis transplanted with *Uch-l1*^+/+^ SSCs collected 2-months post transplantation. (**B**) Representative colony from recipient testis A. (**C**) Recipient testis transplanted with *Uch-l1*^−/−^ SSCs two months post transplantation. (**D**) Representative colony from recipient testis B. Scale bar = 1 mm. (**E**–**H**) Transplanted colony analysis. (**E**) Colony number and (**F**) colony length two months post transplantation (*n* = 7 and 6 for *Uch-l1*^+/+^ vs. *Uch-l1*^−/−^ SSCs transplantation, respectively). (**G**) Colony number and (**H**) total colonized length 10 months post transplantation (*n* = 9 and 6 for *Uch-l1*^+/+^ vs. *Uch-l1*^−/−^ SSCs transplantation, respectively). (**I**) Representative transplanted colony cross-sections of dark (left), medium (middle) and light (right) blue intensity of *Uch-l1*^−/−^SSCs 10 months post transplantation. Scale bar = 100 µm. (**J**) Proportion of colonies with different degrees of differentiation (blue intensity) from *Uch-l1*^+/+^ and *Uch-l1*^−/−^ SSCs 10 months post transplantation. * represents significant differences *p* < 0.05.

Cultured SSCs derived from *Uch-l1^+/+^* or *Uch-l1^−/−^* mice were subsequently transplanted into the seminiferous tubules of busulfan-treated mouse testes. Testes were collected at two or 10 months post-transplantation to determine stem cell number (colony number), stem cell capacity (colony length) and score of a complete spermatogenesis of the transplanted cell population (Figure 6A–D). As illustrated in Figure 6E,F, no differences were found between *Uch-l1^+/+^ and Uch-l1^−/−^* colony numbers or colony length at two months post-transplantation. At 10 months post-transplantation, there was no difference in the colony number between the two genotypes (Figure 6G). Due to the increased length of colonies at the 10-month collection time point, the incidence of merged colonies was high; therefore, individual colony length could not be assessed accurately. When the total length of colonized seminiferous tubules was measured, *Uch-l1^−/−^* recipient testes had significantly higher total colonized tubule length compared to *Uch-l1^+/+^* recipients (Figure 6H). The intensity of the blue X-gal staining was scored in three categories: light, medium and dark blue (Figure 6I), reflecting the number of differentiating germ cell layers [48]. Dark blue colonies had complete donor-derived spermatogenesis, medium blue colonies had fewer germ cell layers and light blue colonies had only one or very few layers of donor-derived cells. The intensity of the blue X-gal staining was lighter in the *Uch-l1^−/−^* colonies than the *Uch-l1 ^+/+^* colonies (represented in Figure 6B,D,J). A larger proportion of *Uch-l1^−/−^* colonies were medium blue and a smaller proportion were dark blue as compared to the *Uch-l1^+/+^* colonies, indicating less differentiation and lower capacity to regenerate full spermatogenesis in *Uch-l1^−/−^* colonies (Figure 6J).

### 3.6. Loss of Regenerative Capacity Is Accompanied with Decreased Mitochondrial Respiration in Cultured Uch-l1^−/−^ SSCs

**Figure 7:**
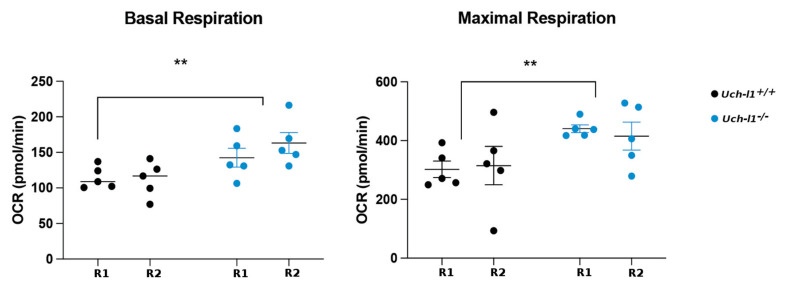
Representative Figure of OCR assessment and quantification of basal, maximal respiration, and respiration used for ATP production per biological replicate (*n* = 10 per biological replicate). ** represents significant differences *p* < 0.01.

SSCs undergo a metabolic switch from glycolysis to OXPHOS with differentiation [49]. Anaerobic metabolism is required for maintenance of stemness in vivo and in vitro and decreased glycolysis is associated with a lower differentiation potential of SSCs [33,49]. Transcriptome data revealing dysregulation of metabolic transitions in *Uch-l1^−/−^* as compared to Uch-l1+/+, together with a decline in spermatogenic differentiation potential of *Uch-l1^−/−^* after transplantation, prompted us to elucidate the impact of UCH-L1 loss on mitochondrial respiration. Two different biological samples each for *Uch-l1^−/−^* and *Uch-l1^+/+^* were assessed with the Seahorse flux analyzer after 21 cell passages (150 days). Similar to the shift observed at the transcriptional level (Figure 4D), we detected significantly higher basal respiration and maximum respiration capacity in *Uch-l1^−/−^* (Figure 7). This underlines that the loss of UCH-L1 altered the metabolic homeostasis of SSCs and therefore stem cell maintenance. It explains the decrease in the SSC differentiation potential, as assessed by scoring of full spermatogenesis described above, and indicates a role for UCH-L1 in maintenance of SSC metabolic integrity and low aerobic metabolism. There was, however, a lack of correlation between the OCR data and OXPHOS-associated gene expression as assessed by qPCR. As shown in Table S2, no significant difference in **Δ**Ct values was observed between the two genotypes, except for the superoxide dismutase (*Sod)1* gene (Table S2). This data is not surprising if a feedback loop leading to mRNA degradation exists when OXPHOS-related proteins are synthesized at high quantities. Collecting samples for qPCR at earlier time points may have provided better insight into the pattern of OXPHOS-associated gene expression in cultured SSCs.

## 4. Discussion

The role of UCH-L1 in the nervous system has become significantly better understood due to the contributions of *Uch-l1* knockout mouse models; however, its function in spermatogonia has not been clearly identified. We report here that the loss of UCH-L1 impacts the differentiation competence of murine SSCs in vivo. The observed disruption of metabolic homeostasis in undifferentiated spermatogonia and loss of anaerobic metabolism of SSCs after long-term cell culture indicate a role of UCH-L1 in the maintenance of low OXPHOS and protection of stemness-associated metabolism in SSCs.

UCH-L1 is highly expressed in undifferentiated spermatogonia and early differentiating spermatogonia, but not in further differentiating germ cells, and this expression pattern is phylogenetically conserved in mouse, pig and human testes [38,50,51]. However, whether UCH-L1 is required for normal spermatogenesis remains poorly understood. We used a *Uch-l1* knockout mouse model to investigate morphological and functional changes associated with UCH-L1 function deficiency.

We report here a significant decrease in testis weight in *Uch-l1^−/−^* compared to *Uch-l1^+/+^* mice at 120 days of age. Testis weight is an important reproductive trait in males that reflects both the spermatogenic capacity, as the sperm production rate positively correlates with the testis weight [52], and the mating potential [53]. Although *Uch-l1^−/−^* mice remained fertile until at least 3 months of age, a reduction in confirmed matings and litters produced was observed in this study. It remains, however, plausible that the reduction in the *Uch-l1^−/−^* mice’ ability or desire to copulate may also have resulted from progressive neurodegenerative hind limb paralysis [54].

Of note, decreased testis weight was accompanied by a significant increase in the number of degenerative seminiferous tubules in *Uch-l1^−/−^* compared to *Uchl-1^+/+^* at 120 day of age. Considering the progressive nature of the seminiferous tubule degeneration, this phenotype becomes more pronounced in *Uch-l1^−/−^* mice with age. Previous findings using the *gad* mouse model corroborate this assumption as a slight decrease in the size of seminiferous tubules was reported in 25-week old homozygous *gad* mice compared to those of age-matched wild-type mice [20]. Since the final 120-day time point was dictated by the strong neurodegenerative phenotype, it was not possible to assess the *Uch-l1^−/−^* germinal epithelium in older animals. *Uch-l1*-deficient Sertoli cells also may lose their ability to provide morphological and nutritional support needed for spermatogenesis, contributing to spermatogenic failure [55,56].

Maintaining a normal seminiferous epithelium relies on a regulated balance between proliferation, differentiation, and apoptosis of germ cells [1]. A potential role of UCHL1-dependent apoptosis in germ cells and sperm maturation has been suggested [30]. Testes in the *gad* mouse model showed resistance to the early and massive wave of germ cell apoptosis during the pre-pubertal stage of development, a phase that is essential for functional spermatogenesis in adults. In contrast, there was no difference in cell apoptosis between *Uch-l1^−/−^* and *Uch-l1^+/+^* mice in the current study, either in situ or in vitro from day 7 to day 120, a timeline that captures the first as well as subsequent spermatogenic waves. The discrepancy between the two studies might be explained by a potential phenotypical difference between the two mouse models. The *gad* model is the result of a spontaneous congenital mutation and might possibly display additional mutations within the ubiquitin–proteasome pathway, while the model used in our study is a targeted mutant mouse model. Nonetheless, our data suggest that the progressive loss in the seminiferous tubule structure might be associated with a decrease in cell proliferation or differentiation rather than an increase in germ cell loss, since no difference in TUNEL-positive cells was apparent between the two genotypes at 90 or 120 days of age.

Long-term transplantation data support these conclusions. At 10 months post transplantation, the colonized area had expanded more in testes that received germ cells from *Uch-l1*^−/−^ mice as compared to *Uch-l1^+/+^*. Interestingly, these *Uch-l1*^−/−^ colonies contained fewer layers of donor-derived cells, indicating that *Uch-l1^−/−^* SSCs had a reduced capacity to regenerate the entire spermatogenic lineage. Our data showing no difference in the cell number, viability, proliferation as well as in spermatogonia-associated gene expression (*Id4*, *Plzf*, c-*Kit* and *Stra8*) between *Uch-l1^+/+^* and *Uch-l1^−/−^* in culture, suggest that the intrinsic loss of spermatogenic potential of *Uch-l1^−/−^* SSCs did not affect normal SSC gene expression and viability.

It is becoming increasingly evident that maintaining metabolic integrity and plasticity is pivotal for SSC function [33,49,57]. Anaerobic metabolism is necessary for SSC maintenance in vivo and in vitro [33,49], while SSCs upregulate mitochondrial activity with differentiation [40,42,49,58]. The protection of this metabolic balance by preserving the metabolic integrity of SSCs is essential to maintaining the differentiation potential of SSCs, which gradually diminishes in aging cells [58].

We show here that *Uch-l1^−/−^* SSCs kept in culture for 21 passages (150 days) had a higher basal respiration and maximum respiration capacity as compared to *Uch-l1^+/+^*, suggesting a loss of stem cell metabolic integrity in UCH-L1-deficient SSCs. These data indicate that the loss of UCH-L1 was detrimental to metabolic homeostasis regulating SSC function, therefore causing a decrease in SSC differentiation competence, all of which was reflected in the long-term outcome after transplantation of *Uch-l1^−/−^*-derived SSCs.

Furthermore, our single-cell RNA sequencing data are consistent with a disturbed shift in cell metabolism, as an upregulation of genes associated with OXPHOS was observed in *Uch-l1^−/−^* SSC/progenitors whereas genes associated with hypoxia were upregulated in *Uch-l1^+^l^+^* SSC/progenitors. Upregulation of genes associated with glycolysis and hypoxia was detected in early differentiating spermatogonia of UCH-1*^−/−^*, as opposed to the physiological switch towards OXPHOS in the wild-type. Hence, UCH-L1 deficiency altered the bioenergetic equilibrium between glycolysis and OXPHOS that is necessary for spermatogonial proliferation and differentiation [58]. These data suggest that UCH-L1 is required for maintenance of proper mitochondrial activity and its upregulation leads to impaired function of SSCs, impairing SSC metabolic and differentiation competence. Single-cell RNA sequencing captured these metabolic perturbations largely in one biological replicate. As these data represent a snapshot of a single time point, it is possible that these metabolic changes might not occur at similar rate or speed in mice, considering variability that might exist between individual mice of the same age group, and the compromising effect that the loss of UCH-L1 may additionally exert on the cellular oxidative defense mechanisms. In fact, UCH-L1 deficiency in the brains of knockout mice was accompanied by decreased levels of glutathione [59], which functions as an antioxidant and redox regulator [60]. The detrimental effect that UCH-L1 loss has on mitochondrial function in cultured SSC aligned with progressive metabolic perturbations that might interfere with SSC differentiation as mice age.

We detected metabolic dysregulation in murine *Uchl1^−/−^* SSCs, characterized by an initial upregulation of OXHPOS-related genes followed by their decrease during differentiation, which is the inverse of the metabolic changes in the wild-type. Furthermore, mitochondrial activity also increased in *Uchl1^−/−^* SSCs after long-term culture, while it remained low in wild-type SSCs. Similar metabolic changes have been described in neuroblastoma cells. A disruption of mitochondrial structure through the reduction of mitofusion 2 (MFN2) levels and an increased oxygen consumption rate were observed upon knockdown of UCH-L1 with shRNA within a few days [61].

Although further experiments are warranted to determine the mechanism by which UCH-L1 deficiency affects germ cell metabolism, one may speculate that UCH-L1 plays a role in regulating the function of mitochondria directly via the turn-over of mitochondrial proteins or indirectly via, among others, interference with the mechanistic target of rapamycin (mTOR) or hypoxia-inducible factor-1α (HIF-1α).

UCH-L1 is associated with mitochondrial quality control by regulating the expression of mitofusin-2 (MFN2), thus affecting several mitochondrial functions, especially ultrastructure in neural cells [61]. It also inhibits mTOR [28], potentially in synergy with PLZF [62], all of which might potentially help maintain the metabolic phenotype of SSCs. Studies also underlined the role of hypoxia-inducible factor (HIF) in regulating the expression of enzymes involved in glycolysis in response to hypoxia [63], but also in reducing metabolite entry into the TCA cycle, affecting OXPHOS as well [64,65]. UCH-L1 stabilizes HIF [66], and its depletion caused a significant decrease in HIF-1α protein level in a 3D culture model [67]. As UCH-L1 deficiency inhibits Retinoic acid (RA)-induced differentiation of neuroblastoma tumor cells by regulating RA-induced AKT and ERK1/2 signaling activation [68], and considering the critical role of RA-stimulated ERK1/2 pathway activity in *Stra8* expression and meiotic progression in fetal germ cells [69], one may speculate that UCH-l1 deficiency can lead to a similar outcome in *Uch-l1^−/−^* spermatogonia.

In summary, UCH-L1 plays a critical role in maintaining SSC differentiation competence in mice, as a dysregulation of normal metabolic transitions associated with SSC maintenance and differentiation occurred in *Uch-l1^−/−^*-derived SSCs in vitro and in vivo. Additional studies are required to decipher the underlying mechanism by which UCH-L1 regulates metabolism in SSCs, yet the current study highlights that stage-dependent metabolic regulation is critical for male germ cell function and reveals a unique role of UCH-L1 in this complex system.

## Data Availability

The data presented in this study are available in the article or its supplementary material.

## Supplementary Materials

The following are available online at https://www.mdpi.com/article/10.3390/cells10092265/s1: Figure S1: Representative UCH-L1^+/+^FACS plot of GFRa1+ and c-KIT+ spermatogonia isolation for single cell RNA sequencing, Figure S2: UCH-L1 expression in mammalian testes, Figure S3: TUNEL analysis, Figure S4: Heat maps of top differentially expressed genes between clusters, Figure S5: Distribution of cell clusters between the replicates projected on UMAP plot, Figure S6: *Uch-l1*^+/+^ and *Uch-l1*^−/−^ spermatogonia in vitro, Table S1: Primers list for qPCR, Table S2: Analysis of OXPHOS-associated gene expression in *Uch-l1^−/−^* SSC vs. *Uch-l1^+/+^* SSC by qPCR.
