# Supplementary material for: Loss of Ubiquitin Carboxy-Terminal Hydrolase L1 Impairs Long-Term Differentiation Competence and Metabolic Regulation in Murine Spermatogonial Stem Cells
Source: Cells. 2021 Aug 31;10(9):2265. doi: 10.3390/cells10092265 (PMC8465610; doi:10.3390/cells10092265)
Supplement: Supplementary file 1 [file cells-10-02265-s001.zip › cells-1271916-supplementary.pdf]

Supplementary Materials

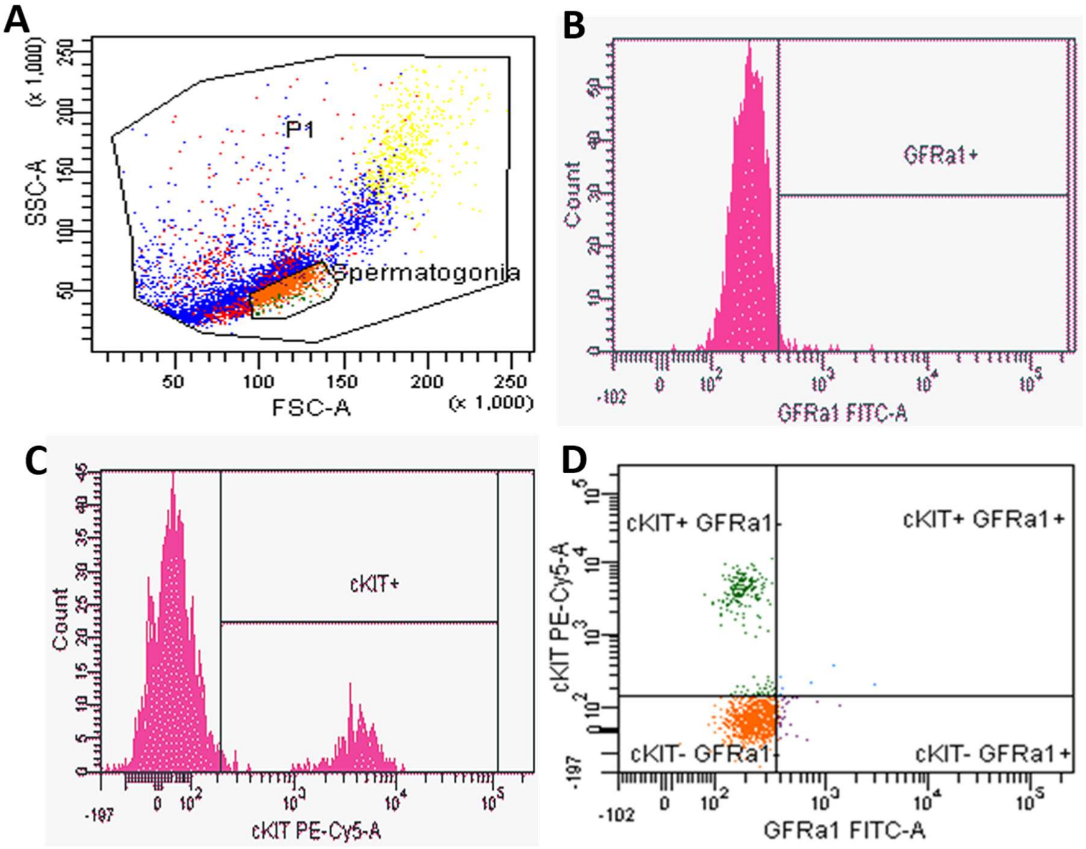

Figure S1. Representative UCH-L1$^{+/+}$ FACS plots of GFRa1+ and c-KIT+ spermatogonia isolation for single cell RNA sequencing. UCH-L1$^{+/+}$ and UCH-L1$^{-/-}$ FACS plots were not distinguishable. (A) Forward and side scatter dot plot of whole testes single cells and gating for spermatogonia enriched population. (B) Histogram of GFRa1+ spermatogonia. (C) Histogram of cKIT+ spermatogonia. (D) Dot plot of GFRa1+ and c-KIT+ spermatogonia for cell sorting. The c-KIT+GFRa1-, c-KIT+GFRa1+, and c-KIT-GFRa1+ populations were isolated collectively for sequencing.

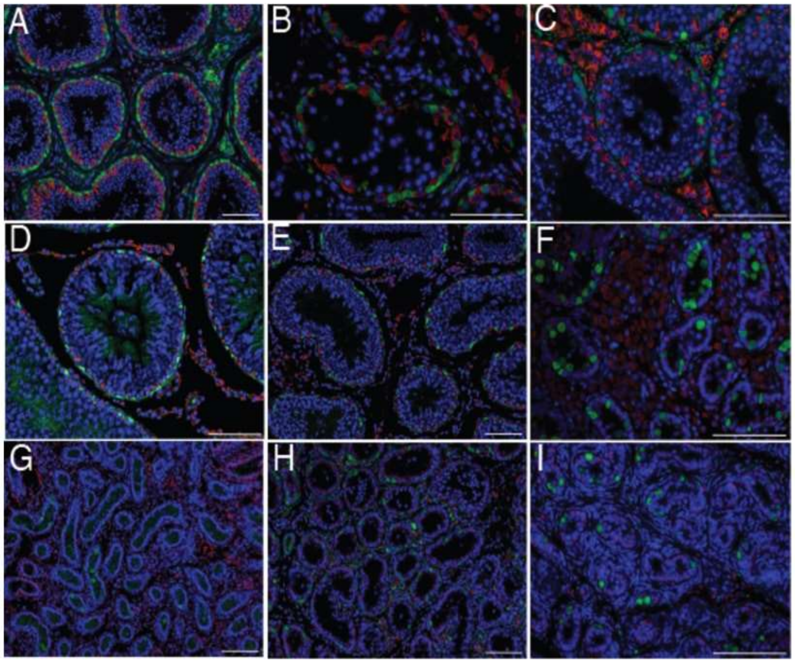

Figure S2. UCH-L1 expression in mammalian testes. Immunohistochemistry of UCH-L1 across mammalian species. (A-I) UCH-L1 = green, Vimentin = red, DAPI = blue and scale bar = 100μm. (A) Adult human testis tissue. (B) Adult buck testis tissue. (C) Adult cat testis tissue. (D) Adult rat testis tissue. (E) Adult sheep testis tissue. (F) Juvenile porcine testis tissue. (G) Juvenile goat testis tissue. (H) Juvenile horse testis tissue. (I) Juvenile monkey tests tissue.

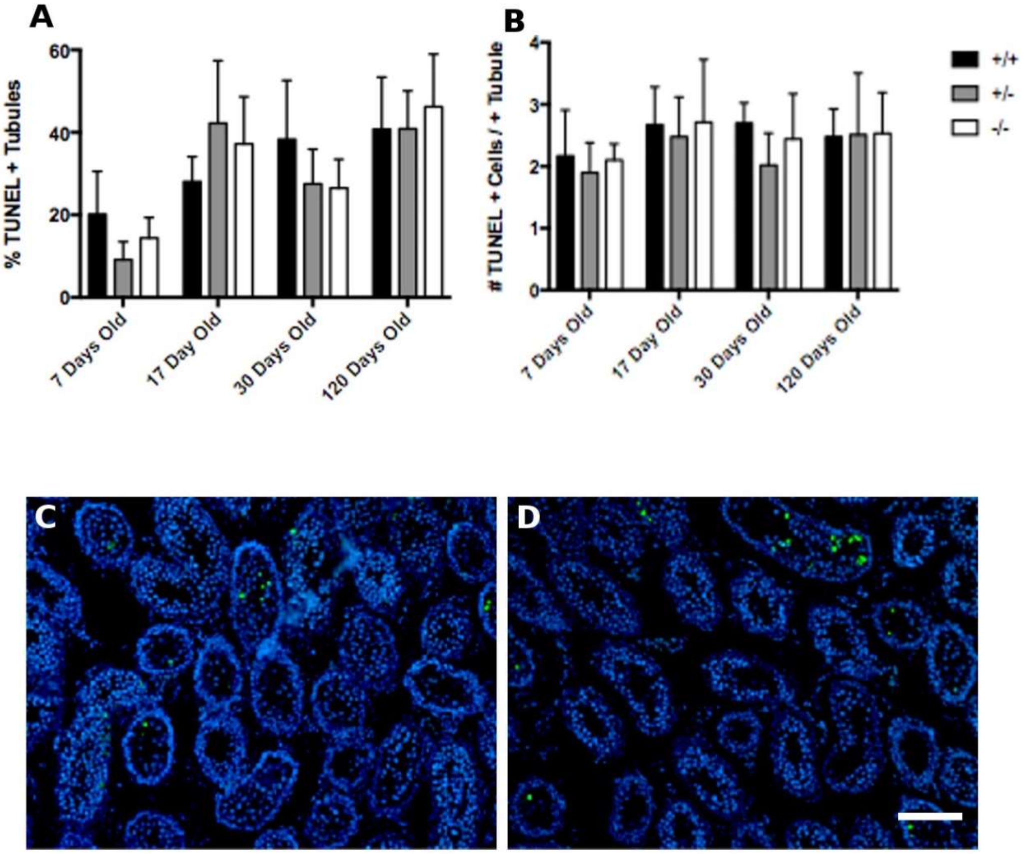

Figure S3. TUNEL analysis. (A-D) TUNEL analysis of *Uch-l1*$^{+/+}$, *Uch-l1*$^{+/-}$ and *Uch-l1*$^{-/-}$ testes. N=6/genotype/time point. (A)Percentage of TUNEL positive tubules at 7, 17, 30 and 120 days old in *Uch-l1*$^{+/+}$, *Uch-l1*$^{+/-}$ and *Uch-l1*$^{-/-}$ testes. (B) Number of TUNEL positive cells per positive tubules at 7, 17, 30 and 120 days old in *Uch-l1*$^{+/+}$, *Uch-l1*$^{+/-}$ and *Uch-l1*$^{-/-}$ testes. (C) Representative image of TUNEL reaction in 17- day old *Uch-l1*$^{+/+}$ testes. (D) Representative image of TUNEL reaction in 17-day old *Uch-l1*$^{-/-}$ testes. Scale bar = 100μm.

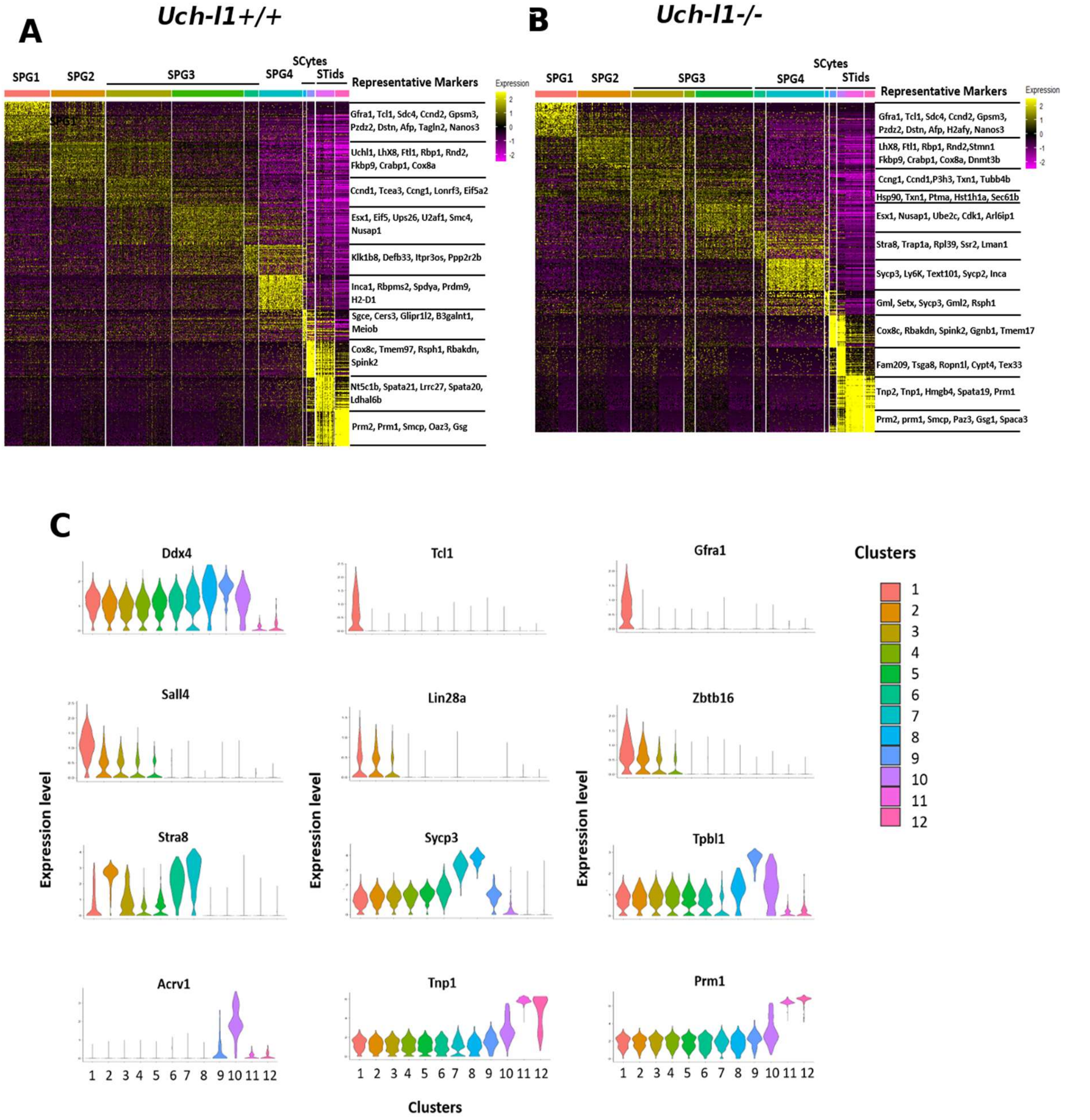

Figure S4. Heat maps of the top differentially expressed genes between clusters. (A) *Uch-l1*$^{+/+}$ (B) *Uch-l1*$^{-/-}$. The top 5 to 10 representative genes selected from each cluster are shown on the right of each heatmap. (C) Violin plots shows the expression of some genes makers that allow the distinction between spermatogonia, spermatocytes and spermatids in 4 merged samples (2 *Uch-l1*$^{+/+}$ and 2 *Uch-l1*$^{-/-}$). The clusters are colored coded.

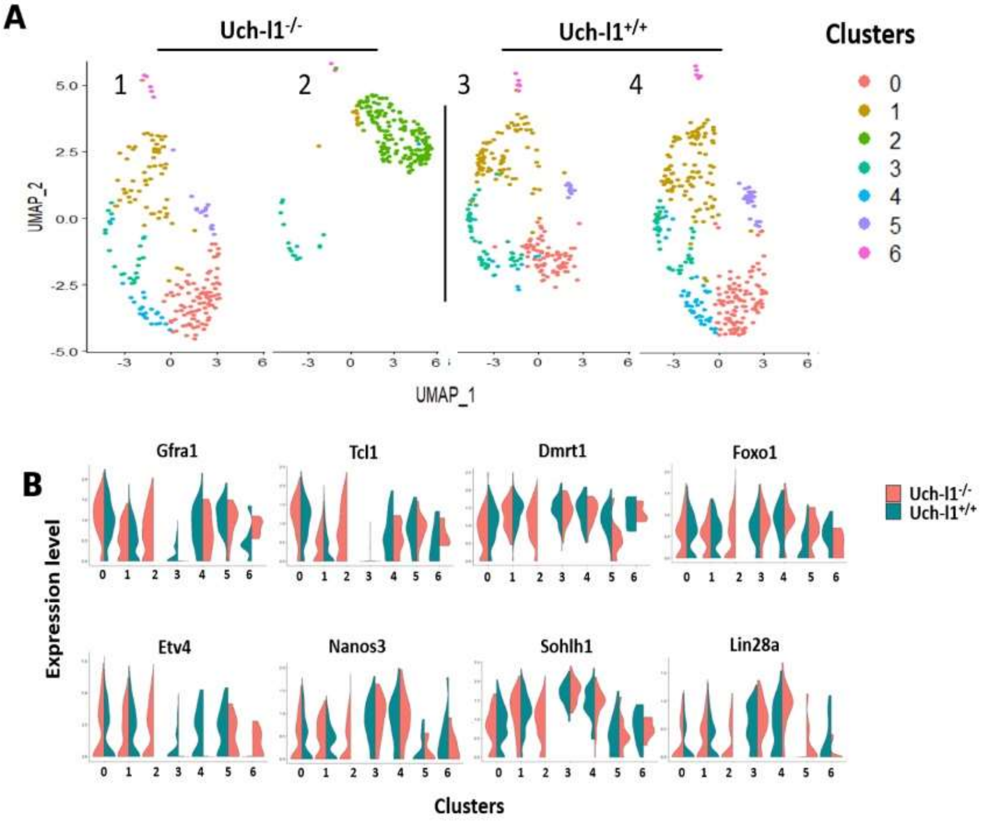

Figure S5. Distribution of cell clusters between the replicates projected on UMAP plot. (A) *Uch-l1*$^{-/-}$ (replicates 1 & 2) and *Uch-l1*$^{+/+}$ (replicates 3 & 4). Cell clusters are colored coded. (B) Violin plots showing comparative analysis of the level of expression of markers specific to SSC/progenitors between *Uch-l1*$^{-/-}$ and *Uch-l1*$^{+/+}$.

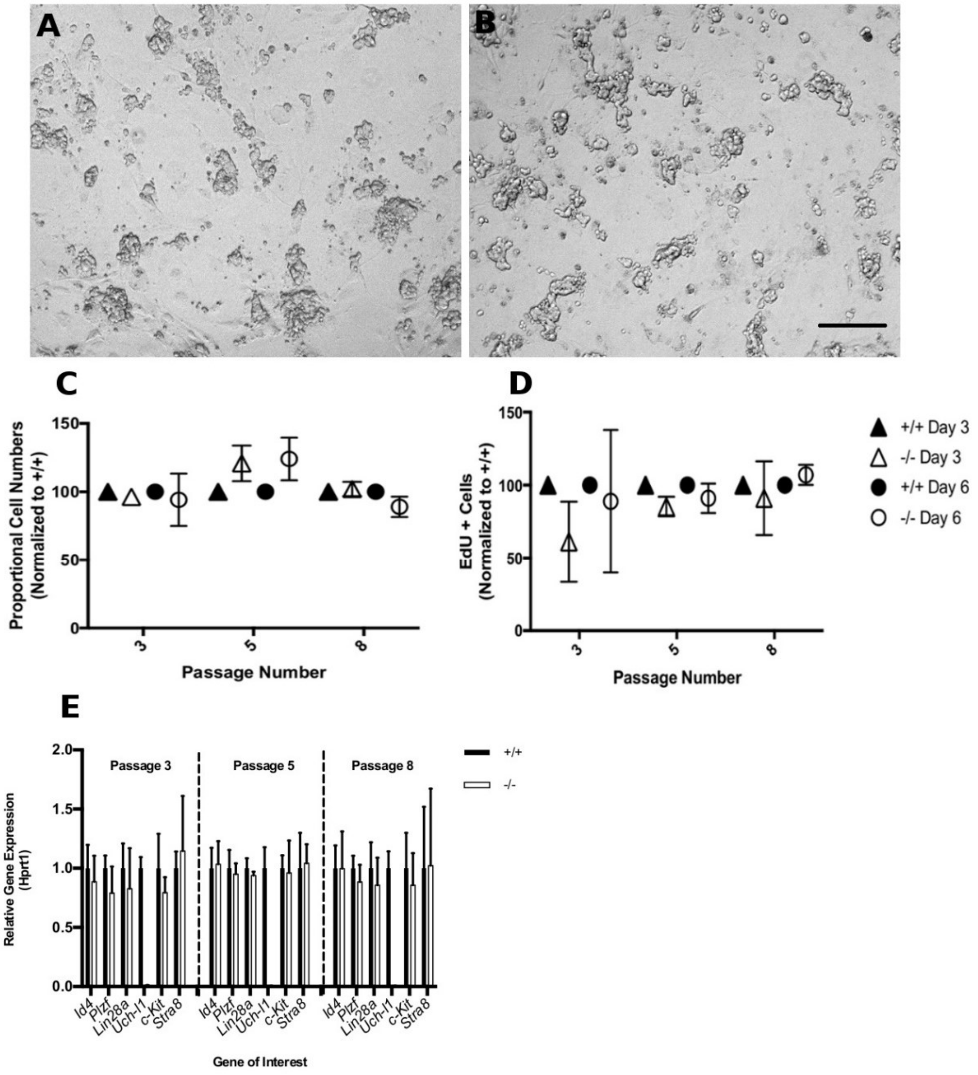

Figure S6. *Uch-l1*$^{+/+}$ and *Uch-l1*$^{-/-}$ spermatogonia in vitro. (A-B) Phase contrast of spermatogonia culture at passage 8, day 3 of culture (*Uch-l1*$^{+/+}$ and *Uch-l1*$^{-/-}$ respectively). Scale bar=100µm. (C-D) Proportional cell number and proliferation of *Uch-l1*$^{-/-}$ spermatogonia cultures compared to *Uch-l1*$^{+/+}$ cultures at passage 3, 5 and 8 on day 3 and 6 of culture. Each passage has 6 independent replicates per genotype. (E) Relative gene expression data of *Uch-l1*$^{+/+}$ and *Uch-l1*$^{-/-}$ spermatogonia cultures at passage 3, 5 and 8 on day 6 of culture. Each passage has 6 independent replicates per genotype with the average of the *Uch-l1*$^{+/+}$ expression being set to 1.

Table S1. Primers list for qPCR.

Genes	Primers
Id4	3′-ACT CAC CGC GCT CAA CAC T-5′<br>5′-GCA CAC CTG GCC ATC CAT-3′
Plzf	3′-TCA TTC AGC GGG TGC CAA AG-5′<br>5′-AGC CAT GTC CGT GCC AGT A-3′
Lin28a	3′-AGG AGA CAG GTG CTA CAA CTG C-5′<br>5′-TGG ACA CGA GGC CAC CAT ATG-3′
c-Kit	3′-CCT CAG CCT CAG CAC ATA GC-5′<br>5′-CTG GCG TTC ATA ATT GAA GTC ACC-3′
Stra8	3′-CTC AAA GCA TCC TTC AAC CTG CA-5′<br>5′-AGG AAT ACA CTG TCA TTC TCG GAA -3′
Uch-l1	3′-GCT GGA ATT TGA GGA TGG ATC CGT -5′<br>5′-CTG GAT GGC CTC GTT CTT CTC G-3′
Hprt1	3′-AGC AGT ACA GCC CCA AAA TGG T-5′<br>5′-CCA ACA AAG TCT GGC CTG TAT CC-3′
Cox7A	3′-CAC GTC CTT GGG CGA AGA G-5′<br>5′-AGA GCC TGG GAG ACC CGT AG-3′
Ndufa2	3′-TGC TAA CAA AGA TGG CGG CT-5′<br>5′-TGC ACG ATG AAA TCC CTC ACA-3′
Ndufs4	3′-GTT GGG CAT CAA CCG CTG AC-5′<br>5′-GAT GTA GCC AGC TCC AAC CT-3′
Uqcrc1	3′-TGA GAA CGT CAG GAG GCT GT-5′<br>5′-CGA CAT GGA GTG AGA CCA GG-3′
Uqcr10	3′-CAT CAA CGA GGG GAA ACT GTG-5′<br>5′-GTC GGT GAA CGG CAA CTT GA-3′
Ndufb9	3′-TCA TGG TGT ATC CAC AGG GAC AAG-5′<br>5′-AGC TGG GTA GCC CTC ATC ATA-3′
Cox6b	3′-GTT GGC AGA ACT ACC TGG ACT T-5′<br>5′-CAC ACA CGG AGA CAT CAC CC-3′
Cyc1	3′-CAG CTT CCA TTG CGG ACA C-5′<br>5′-AAA CCC CTC CGA ATG CTG G-3′
Sod1	3′-GTC GGC TTC TCG TCT TGC TC-5′<br>5′-CTG GTT CAC CGC TTG CCT TC-3′
Sod2	3′-GTG GAG AAC CCA AAG GAG AGT T-5′<br>5′-GGT AGT AAG CGT GCT CCC AC-3′
Cat	3′-GAG GGA TTC CCG ATG GTC AC-5′<br>5′-TGC CCT GGT CGG TCT TGT AA-3′
Cox5a	3′-TTG CGA GCA TGT AGA CGG TT-5′<br>5′-GTC CTT AGG AAG CCC ATC GAA-3′
Atp5a	3′-CAG GAG AGG GGC GGT ACT T-5′<br>5′-AAA GCA TTT TTG GAG ACC AGT CC-3′
Ndufa1	3′-TTG GCG TGC GAG TAA CGG-5′<br>5′-AGC AAC TCG TTT TTC CTT GCC C-3′
Ndufa3	3′-ACC CTA CAA CTA CCC AGT GCC-5′<br>5′-AGG TTC TTC AGC CAG TCC AAG-3′
Ndufa5	3′-AGG ACG TTG GTG TAG AGG ACA-5′<br>5′-CCA GAA GAC CAC GCA TCT GT-3′
Cox6c	3′-CAT TTC TTC CGC CTT CCG TG-5′<br>5′-AAA ATG GCA AAT TCT TCC CCG-3′
Sdha	3′-ACT GTT ATT GCT ACT GGG GGC-5′<br>5′-CTT CTG TGA TGA GGC AGC CA-3′
Cox7c	3′-CGG GGA AGA ATT TGC CAT TTT CAG TGG-5′<br>5′-AAA GAA AGG TGC GGC AA ACC-3′
Uqcr11	3′-CTG CAC ATG CGT AGT GCT CC-5′<br>5′-GGC TGT GGG AAT CCA GTT T-3′

Table S2. Analysis of OXPHOs-associated Genes expression in *Uch-l1*$^{-/-}$ SSC *vs. Uch-l1*$^{+/+}$ SSC by qPCR

Gene	***Uch-l1*** **+/+ Δct**	***Uch-l1*-/- Δct**	**Fold Change**
Cox7a	4.11 ± 0.13	3.69 ± 0.05	1.037677914
Ndufa2	4.79 ± 0.02	5.32 ± 0.004	0.654383153
Ndufs4	4.12 ± 0.01	4.53 ± 0.05	0.789418204
Uqcr1	4.44 ± 0.04	4.85 ± 0.002	0.755516834
Uqcr10	8.49 ± 0.02	9.04 ± 0.13	0.686230397
Ndufb9	1.09 ± 0.01	1.24 ± 0.004	0.904399531
Cox6b	0.76 ± 0.08	0.78 ± 0.02	0.986297206
Cyc1	3.22 ± 0.005	3.75 ± 0.04	0.693887618
Cox6c	0.39 ± 0.35	0.21 ± 0.06	1.134716167
Cox7c	—0.98 ± 0.02	—0.96 ±0.03	0.990357386
Cox5a	1.93 ± 0.01	2.07 ± 0.02	0.907742808
Atp5a	10.68 ± 0.06	11.51 ± 0.05	0.562674034
Ndufa1	2.85 ± 0.02	2.87 ± 0.006	0.989797965
Ndufa3	3.14 ± 0.02	3.05 ± 0.04	1.061058695
Sod1	*1.37 ± 0.66*	*—0.2 ±0.36*	*3.07179029*
Sod2	2.19 ± 0.22	1.96 ± 0.02	1.170111201
Cat	1.89 ± 0.14	1.92 ± 0.14	0.983145641
Sdha	5.26 ± 0.001	5.06 ± 0.11	1.156593488
Uqcr11	6.32 ± 0.006	6.00 ± 0.03	1.242051804
Ndufa5	—1.18 ± 0.03	—1.33 ± 0.05	1.115662955
